# Accidental Foreign Body Aspiration Through Tracheostomy Inlet; 26 cases

**DOI:** 10.34172/aim.2022.50

**Published:** 2022-05-01

**Authors:** Aykut Eliçora, Hüseyin Fatih Sezer, Galbinur Abdullayev, Adil Avcı, Salih Topçu

**Affiliations:** ^1^Department of Thoracic Surgery, Faculty of Medicine,Kocaeli University, Kocaeli, Turkey; ^2^Department of Thoracic Surgery, Kocaeli State Hospital, Kocaeli, Turkey

**Keywords:** Aspiration, Foreign Body, Tracheostomy

## Abstract

**Background:**

Foreign body aspiration from tracheostomy is very rare, and materials related to tracheostomy are usually aspirated. This condition, which can lead to serious complications, can be treated using bronchoscopic procedures. In this study, we aimed to present our clinical experience in foreign body aspiration via tracheostomy.

**Methods:**

Data from 26 patients who presented to our hospital for foreign body aspiration via tracheostomy from 2006 to 2020 were analyzed retrospectively.

**Results:**

Foreign bodies were removed by fiber optic bronchoscopy in 15 (57.7%) cases, by rigid bronchoscopy in 9 (34.6%) cases and both methods were used in 2 (7.7%) cases. During bronchoscopy, local anesthetic procedures were used in 13 (50%) cases and general anesthesia was used in 11 (42.3%) cases. No anesthesia was used in two (7.7%) patients who underwent bronchoscopy under intensive care conditions. While the mean operative time for flexible bronchoscopy was 8.77±0.83 (CI: 26.03–29.43) minutes, the mean operative time for rigid bronchoscopy was 27.73±2.53 (CI: 26.03–29.43) minutes.

**Conclusion:**

Both rigid bronchoscopy and fiberoptic bronchoscopy (FOB) have advantages and disadvantages in foreign body removal. In our opinion, it is more reasonable to perform fiber optic bronchoscopy first in patients with a tracheostoma. In the light of our experiences, fiber optic bronchoscopy does not require general anesthesia and the operation time is shorter than rigid bronchoscopy. This feature makes fiber optic bronchoscopy advantageous.

## Introduction

 Accidental tracheobronchial foreign body aspirations were commonly seen in the early childhood, especially frequent in the first three years of life. They are very rare in the adult age group. Neurological diseases affecting swallowing functions, loss of consciousness, alcohol use, maxillofacial trauma, and loss of cough reflex are risk factors that increase tracheobronchial aspiration in adults.^1^ In addition to these risk factors, entry of foreign bodies to the tracheobronchial system becomes much easier since the airway protective mechanism of larynx is impaired after tracheostomy.^2^ Foreign body aspiration from tracheostomy is very rare, and materials related to tracheostomy are usually aspirated. This condition, which can lead to serious complications, can be treated using bronchoscopic procedures.

 There are a few case reports in the literature on foreign body aspirations from tracheostomy, and there are no large series. In this study, we aimed to present our clinical experience in 26 cases with foreign body aspiration through tracheostomy.

## Materials and Methods

 Data from 26 patients who presented to our hospital for foreign body aspiration after tracheostomy between the years 2006 and 2020 were analyzed retrospectively. Chest x-rays and thoracic computer tomography (CT) scans were used for radiologic evaluation of the patients.

 Both flexible and rigid bronchoscopes were used for foreign body removal. All bronchoscopic procedures were performed under operating room conditions. Aerosol lidocaine (max 8.2 mg/kg) as a local anesthetic and intravenous midazolam (0.06–0.07 m /kg) for sedation were administered before a flexible bronchoscopy procedure (Karl Storz Instruments, Germany). Rigid bronchoscopy (Karl Storz Instruments, Germany) procedures were performed under general anesthesia. Foreign bodies seen on bronchoscopic examination were removed with the help of forceps. All bronchoscopic procedures were performed via the tracheostomy tract.

 The cases were evaluated in terms of age, gender, physical examination, medical history, symptoms, admission time to the hospital, cause of tracheostomy, duration of tracheostomy, type of anesthesia administered, nature and localization of foreign body, bronchoscopic procedure type, Glasgow Coma Scale (GCS), operation time, discharge time, radiological examination and radiological findings.

###  Statistical Analysis

 All statistical analyses were performed using IBM SPSS for Windows version 20.0 (SPSS, Chicago, IL, USA). Continuous variables were presented with mean ± standard deviation and median values (25th-75th percentile). Comparison of continuous variables between groups was carried out using Mann-Whitney U test. All statistical analyses were carried out with 5% significance, and a two-sided *P *value < 0.05 was considered as statistically significant. The confidence interval was calculated in numerical values. Since the sample size of our study was small, nonparametric tests were used for comparisons.

## Results

 A total number of 26 cases were included in our study; 21 (80.8%) of these cases were male and 5 (19.2%) were female. The mean age of the cases was 62.19 ± 12.20 years (range of 28 to 82 years). The mean age was 63.43 ± 10.13 (range of 47 to 82 years) in males and 57.00 ± 19.35 (range of 28 to 75 years) in females. No statistically significant difference was found between the male and female groups in terms of age distribution (*P* = 0.659).

 All cases had a tracheostomy. The reasons for tracheostomy administration were as follows: 17 cases (65.4%) had laryngeal carcinoma, 2 cases (7.7%) had prolonged intubation, 2 cases (7.7%) had adenoid cystic carcinoma, 3 cases (11.5%) had tracheal stenosis, 1 case had burns on the neck and 1 case (3.8%) had neurological hypoxia. The mean time of performed tracheostomy was 3.74 ± 2.50 years (range from 0.09 to 10 years) ago. The mean duration of tracheostomy was 6.25 ± 3.86 (range of 1 to 10 years) years in two patients who aspirated cannula from tracheostomy. There was no statistically significant difference between the other aspirations in terms of tracheostomy duration time (*P* = 0.112).

 In the medical history of the cases, 6 patients (23.1%) had chronic obstructive pulmonary disease, 17 (65.4%) had laryngeal carcinoma, 1 (3.8%) had Parkinson’s disease, 2 (7.7%) had tracheal tumor, 6 (23.1%) had cerebrovascular disease, 2 (7.7%) had a myocardial infarction and 1 (3.8%) had a traffic accident (Table 1). The GCS of the cases was determined as 3 in 2 (7.7%) cases, 13 in 1 case (3.8%), and 15 in 23 (88.5%) cases.

**Table 1:** Medical History of Patients

**History**	**Number of Patients (%)**
Chronic obstructive pulmonary disease	6 (23.1)
Laryngeal carcinoma	17 (65.4)
Tracheal tumor	2 (7.7)
Parkinson’s disease	1 (3.8)
Cerebrovascular disease	6 (23.1)
Myocardial infarction	2 (7.7)
Traffic accident	1 (3.8)

 The most common symptom was cough among 13 (50%) cases. The other common symptoms were dyspnea and hemoptysis. No symptoms were observed in 6 (23.2%) of the cases (Table 2).

**Table 2:** Physical Examination Signs and Symptoms

	**Number of patients (%)**
**Physical examination**	
Stridor	5 (19.2)
Wheezing	5 (19.2)
Decreased breathing sounds	1 (3.8)
Decreased saturation	2 (7.7)
High mechanical ventilator pressure	2 (7.7%)
No finding	13 (50)
**Symptom**	
Cough	13 (50)
Dyspnea	9 (34.6)
Hemoptysis	2 (7.7)
Asymptomatic	6 (23.2)

 On physical examination, there was no finding in 13 (50%) cases, stridor in 5 (19.2%) cases, wheezing in 5 (19.2%) cases, decreased breathing sounds in 1 (3.8%) case, decreased saturation in 2 (7.7%) cases and 2 cases (7.7%) had high mechanical ventilator pressure (Table 2).

 The mean time of admission to the hospital after foreign body aspiration was found to be 13.81 ± 15.28 (range 2 to 72) hours.

 A chest x-ray was performed for radiological examination in all cases. Computerized tomography of thorax was performed in 13 (50%) cases. On radiological evaluation, 19 (73.1%) of the foreign bodies were detected directly; in 4 patients (15.4%), a significant difference of ventilation between the two hemithoraces was detected as an indirect finding. No radiological finding was detected in seven (26.9%) of the cases.

 Foreign bodies were removed by fiber optic bronchoscopy in 15 (57.7%) cases, rigid bronchoscopy in 9 (34.6%) cases and both procedures in 2 (7.7%) cases. During bronchoscopy, local anesthesia was used in 13 (50%) cases and general anesthesia in 11 (42.3%) cases. No anesthesia was used in two (7.7%) patients who required bronchoscopy under intensive care unit conditions.

 The mean procedure time was 8.77 ± 0.83 (CI: 26.03-29.43) minutes in patients treated with flexible bronchoscopy and 27.73 ± 2.53 (CI: 26.03-29.43) minutes in patients treated with rigid bronchoscopy (Table 3). The procedure time proved to be statistically significantly different between the two groups (*P* = 0.016).

**Table 3:** Comparison of Flexible and Rigid Bronchoscopy Operation Time

	**Flexible Broncoscopy**	**Rigid** **Broncoscopy**	***P *** **Value**
Mean ± SD (95% CI)	8.77 ± 0.83 (8.27–9.27)	27.73 ± 2.53 (26.03–29.43)	0.016^*^
Minute	9	26	
	8	27	
	9	26	
	9	26	
	9	26	
	8	29	
	11	27	
	7	26	
	9	32	
	9	33	
	9	27	
	8		
	8		
	8		
	9		

SD, Standard deviation; CI, Confidence interval. ^*^Mann-Whitney U Test.

**Figure 1:**
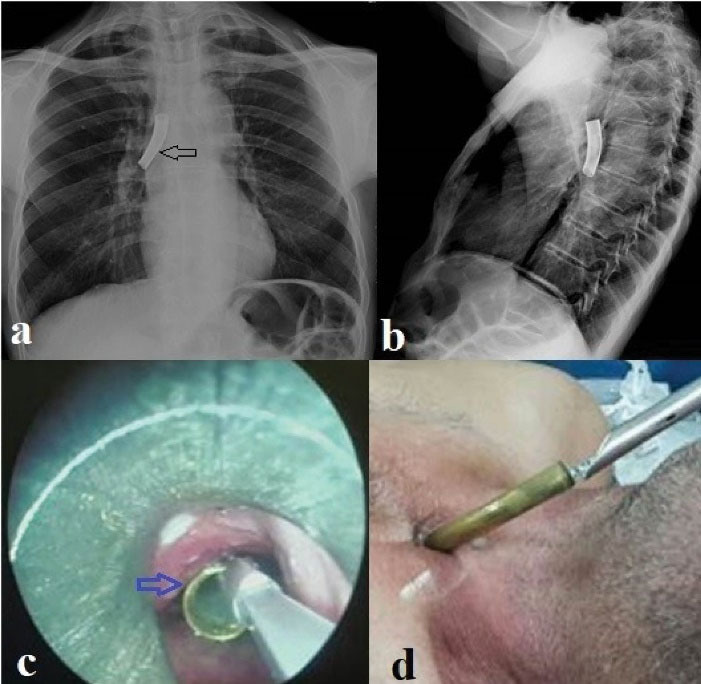
Removal of a patient who aspirated the tracheostomy cannula with a rigid bronchoscope.** (a)** Posterior-Anterior (PA) chest x-ray; black arrow shows the tracheostomy cannula in the right main bronchus. (**b) **View of the tracheostomy cannula on lateral chest radiography. (**c and d) **Rigidbronchoscopy, blue arrow indicates intraoperative tracheostomy cannula.

**Figure 2:**
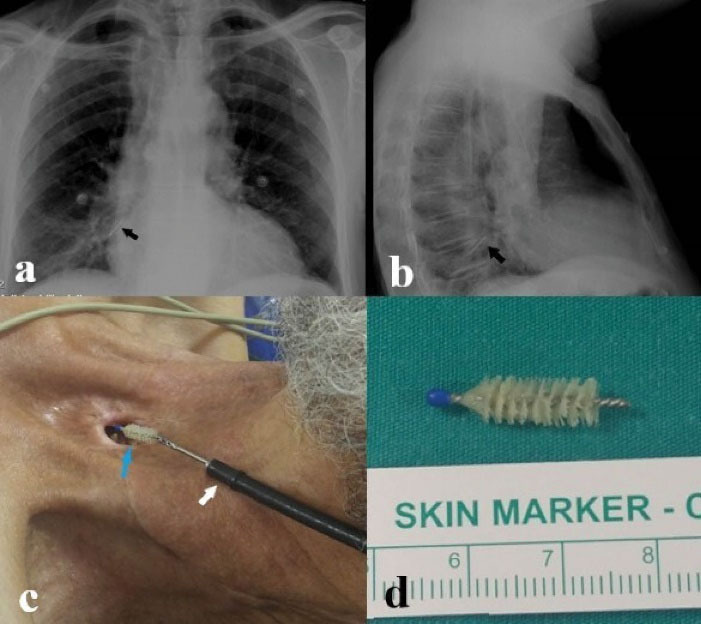
View of voice prosthesis cleaning brush removed with fiberoptic bronchoscope under local anesthesia.** (a and b) **Posterior-anterior and lateral chest x-ray; black arrow indicates cleaning brush in the right bronchus. (**c) **White arrow indicates the fiberoptic bronchoscope, blue arrow shows the image of the cleaning brush as it is being removed. (**d) **Voice prosthesis cleaning brush.

**Figure 3:**
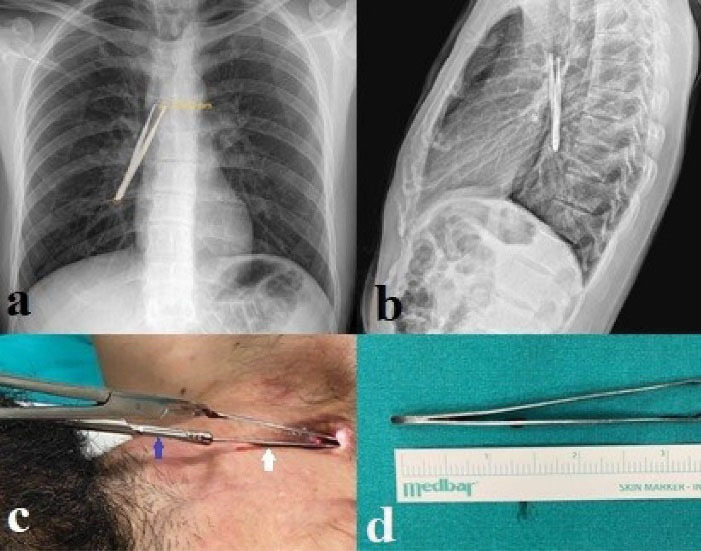
Intraoperative view of tweezers, a very rare foreign body aspiration.** (a and b) **Posterior-anterior and lateral chest x-ray; showtweezers.** (c) **White arrow indicates foreign body (tweezers), blue arrow shows the rigid bronchoscopic forceps. (**d) **Tweezers.

 Foreign bodies were evaluated in terms of location as follows: 11 foreign bodies (42.4%) were in the trachea, 9 (34.6%) in the right main bronchus and 6 (23%) in the left main bronchus. Foreign bodies types were as follows: Vocal prosthesis in 10 (38.5%) cases, tracheostomy tube in 4 (15.4%) cases (Figure 1), cleaning brush in 4 (15.4%) cases (Figure 2), aspiration catheter in 2 (7.7%) cases, plastic plug in 2 (7.7%) cases, pencil cap in 2 (7.7%) cases, tweezers in 1 (3.8%) case (Figure 3) and plastic rod in 1 (3.8%) case.

 The mean patient discharge time was 8.76 ± 7.25 (CI: 4.38–13.15) hours in the flexible bronchoscopy group and 15.00 ± 8.51 (CI: 9.28–20.72) hours in the rigid bronchoscopy group. Although there was no statistically significant difference between the two groups in terms of discharge time, there was a large numerical difference (*P* = 0.119). Two patients who underwent flexible bronchoscopy in the intensive care unit were not included in the evaluation because the discharge time was too long for other reasons (Table 4).

**Table 4:** Comparison of the Discharge Time of Patients who Underwent Flexible and Rigid Bronchoscopy

	**FlexıbleBroncoscopy**	**Rıgıdbroncoscopy**	***P*** ** value**
Mean ± SD (95% CI)	8.76 ± 7.25 (4.38-13.15) h	15.00 ± 8.51 (9.28-20.72) h	0.119^*^
Hour	3	24	
	4	24	
	6	16	
	a	12	
	12	18	
	3	17	
	5	22	
	4	20	
	3	16	
	3	24	
	a	24	
	4		
	5		
	3		
	7 h		

SD, Standard deviation. CI, Confidence interval. *Mann-Whitney U Test. ^a^Intensive care patients undergoing fiberoptic bronchoscopy (Excluded from the study).

## Discussion

 Tracheobronchial foreign body aspiration is a life-threatening emergency. Although it is commonly seen in the pediatric age, it can occur in all age groups.^1-3^ Foreign body aspirations in adults increase with age.^3^ The incidence of cases has been shown to be particularly higher in the population over 75 years of age. This increase is thought to be because of additional diseases such as neurological diseases that occur with aging. The mean age in our cases was 62.19 ± 12.20 years. In addition to all these risk factors, the risk of foreign body aspiration during tracheostomy increases when the protective mechanisms of the tracheobronchial system are impaired. In particular, when tracheostomy and stoma are performed after laryngectomies, the risk of foreign body aspiration increases significantly.^1,4^ Deep inspiration or coughing, together with the vacuum effect created around the stoma, lead to foreign body aspiration.^4^ In our cases, the most common reason for tracheostomy was laryngectomy due to laryngeal cancer. Therefore, in our study, the rate of male patients was higher than women.

 The type of tracheobronchial foreign bodies may vary depending on age, geographic location, socioeconomic level, daily lifestyle, and religious beliefs.^5^ The most common tracheobronchial foreign bodies are organic foreign bodies such as hazelnut, peanut, watermelon seed, chestnut, corn, chickpea and nutshell.^3,6^ Many inorganic foreign bodies such as toy parts and pencils are also commonly aspirated into the tracheobronchial system. Organic foreign bodies, especially foreign bodies such as legumes or corn, enlarge with secretions and can cause total airway obstruction. Foreign bodies such as a battery can cause early mucosal necrosis.^7^

 The type of foreign body aspirated from the tracheostomy differs from other tracheobronchial foreign bodies. The most common foreign bodies in our study were voice prosthesis and cleaning brush (Table 5). The tracheostomy cannula was totally aspirated in four cases. In our study, it was observed that patient education and the medical materials used are very important factors that contribute to foreign body aspiration from tracheostomy. Educating the patient about the care of the voice prosthesis and on-time replacement of the voice prosthesis are important factors that prevent aspiration from tracheostomy. Giving a single cleaning brush alongside with the patients having voice prosthesis causes the use of this brush for a long time. Cleaning brushes used for a long time are broken and aspirated. Similarly, metal cannulas that are not changed for a long time can be broken and aspirated, as well.^8^

**Table 5:** General Characteristics

**Age**	**Gender**	**Tracheotomy Etiology**	**Tracheotomy Time**	**Foreign Body**	**Bronchoscopic Procedure**	**Localization**
67	M	Larynx cancer	2 years	Voice prosthesis	Fiberoptic	Right main bronchus
59	M	Prolonged ıntubation	30 days	Aspiration catheter	Fiberoptic	Trachea
71	M	Larynx cancer	1 year	Cleaning brush	Fiberoptic	Left main bronchus
62	M	Larynx cancer	3 years	Cleaning brush	Fiberoptic	Right main bronchus
70	M	Larynx cancer	4 years	Plastic plug	Rigid	Trachea
75	M	Larynx cancer	6 years	Pencil cap	Rigidand fiberoptic	Right main bronchus
58	M	Larynx cancer	2 years	Voice prosthesis	Fiberoptic	Trachea
47	M	Adenoid cystic carcinoma	3 years	Voice prosthesis	Fiberoptic	Trachea
47	F	Prolonged ıntubation	40 days	Aspiration catheter	Fiberoptic	Trachea
64	M	Larynx cancer	8 years	Tracheostomy cannula	Rigid	Right main bronchus
79	M	Larynx cancer	5 years	Voice prosthesis	Fiberoptic	Right main bronchus
28	F	Adenoid cystic carcinoma	2 years	Voice prosthesis	Fiberoptic	Trachea
50	M	Larynx cancer	4 years	Plastic plug	Rigid	Trachea
63	M	Larynx cancer	6 years	Cleaning brush	Fiberoptic	Left main bronchus
49	M	Larynx cancer	3 years	Voice prosthesis	Fiberoptic	Right main bronchus
82	M	Larynx cancer	4 years	Voice prosthesis	Rigid	Left main bronchus
65	F	Tracheal stenosis	1 year	Tracheostomy cannula	Rigid	Left main bronchus
77	M	Larynx cancer	6 years	Tracheostomy cannula	Rigid	Trachea
53	F	Neurological disease (hypoxia)	3 years	Plastic stick	Rigid	Trachea
76	M	Larynx cancer	7 years	Voice prosthesis	Fiberoptic	Trachea
70	F	Tracheal stenosis	2 years	Voice prosthesis	Fiberoptic	Left main bronchus
25	M	Burns on the neck	3 years	tweezers	Rigid	Right main bronchus
62	M	Tracheal stenosis	1 year	Pencil cap	Rigid and Fiberoptic	Left main bronchus
55	M	Larynx cancer	6 years	Cleaning brush	Fiberoptic	Right main bronchus
61	M	Larynx cancer	10 years	Tracheostomy cannula	Rigid	Trachea
71	M	Larynx cancer	5 years	Voice prosthesis	Fiberoptic	Right main bronchus

M, Male; F, Female.

 The symptoms of the patients after aspiration are quite variable. Some cases are asymptomatic in the early period, while respiratory arrest may develop in other cases.^9^ The most important factors in the variability of symptoms are the localization and type of the foreign body. In the early period, dyspnea, wheezing, and cough are the most common symptoms.^1,3,9^ In addition to these symptoms, stridor, hoarseness, increased oral secretion, increased respiratory effort and agitation may be observed. The most common symptoms in our patients were cough and dyspnea. In our patients, six cases were asymptomatic.

 Early diagnosis and treatment is very important in foreign body aspiration. Patients who are not correctly diagnosed may receive incorrect long-term treatment due to misdiagnosis, such as allergic asthma and lower respiratory tract infection. Untreated foreign bodies in the early period can lead to granulation tissue in the respiratory tract, recurrent lung infections, hemoptysis, lung abscess, bronchiectasis, bronchial stenosis, tracheal lacerations, and fistulas.^4,10^ In foreign body aspirations from tracheostomy, patients are treated earlier than other tracheobronchial foreign body aspirations because they notice the foreign body earlier. In our study, the mean time of admission to the hospital was 13.81 hours (min: 2; max: 72 hours).

 Chest x-ray and computerized tomography of thorax (CT) are the most commonly used radiological examinations in foreign body aspiration. Radiopaque foreign bodies can be seen directly in radiological examinations. Indirect findings such as air trapping, mediastinal shift, emphysema, and atelectasis can be seen in non-radiopaque foreign bodies.^11^ Some studies have reported that no findings were found on chest radiograph in patients with foreign body aspiration.^12^ In our study, all cases were evaluated with chest radiographs. The foreign body was observed in 26.9% of the cases evaluated only by chest radiography. A radiopaque metallic body was observed in the evaluation of PA chest radiographs of our patients who aspirated the tracheostomy cannula. In our patients who aspirated cleaning brushes, only metallic parts of the brushes were opaque, which could be partially evaluated on PA chest radiography (Figure 2). Indirect findings were observed in 15.4% of the chest radiographs. Air trapping was the most common inductive finding. Thorax CT is the radiological examination with the highest diagnostic value in foreign body aspiration. In our study, the localization of the foreign body was detected in all cases evaluated with thorax CT. Since the prosthesis was partially opaque in our patients who aspirated a voice prosthesis, no finding was observed on the posteroanterior chest radiograph in most cases. Voice prostheses were observed as opaque foreign bodies on thorax computed tomography.

 Foreign bodies are mostly localized in the right main bronchus because the right main bronchus is larger and more right-angled than the left main bronchus.^13^ In this study, contrary to the literature, foreign bodies were often localized in the trachea. This is due to the size of the aspirated foreign body and the width of the stoma. Due to a large stoma, foreign bodies that cannot be aspirated from the normal tracheobronchial system can be aspirated and are mostly localized at the level of the trachea.

 Although bronchoscopy is the most common treatment method in foreign body aspiration, thoracotomy may be required in some cases.^14^ Rigid bronchoscopy is the most commonly used bronchoscopic treatment method. However, some studies report the use of fiberoptic bronchoscopy (FOB).^15^ Dikensoy et al referred to fiber optic bronchoscopy for foreign body aspiration; they reported that the procedure was performed with a success rate of 83.6% in a total of 457 patients.^15^ According to some authors, FOB should be the only method for removing foreign bodies, since rigid bronchoscopy cannot be performed through the tracheostomy opening.^16^ In our study, FOB was performed in 65.4% of the patients. In 11.5% of our cases, the foreign body was removed by passing the FOB through the rigid bronchoscope. Foreign bodies were completelyre moved in all our cases.

 We believe that the successful use of FOB in foreign body aspiration from tracheostomy was due to the fact that it passes easily through the tracheostomy tract and obstacles such as vocal cords which are not encountered during foreign body removal. According to Dikensoy et al,^15^ FOB is a faster method than rigid bronchoscopy in removing foreign bodies from the tracheobronchial system, and the mean operation time is 10 minutes. In our study, the mean operation time for FOB was 8.77 ± 0.83 minutes, while the mean operation time for rigid bronchoscopy was 27.73 ± 2.53 minutes.

 Different experiences have been reported in the literature regarding the type of anesthesia to be used during bronchoscopy. Some studies advocate that all bronchoscopic procedures should be performed under general anesthesia.^16^ In our study, general anesthesia was used for all rigid bronchoscopy procedures. Sedation was used for FOB. No complications occurred during and after the procedures. In our study, the mean discharge time for patients who underwent local anesthesia was 8.76 ± 7.25 hours, while the discharge time for patients under general anesthesia was 15.00 ± 8.51 hours. According to this result, the type of anesthesia influences the length of the patient’s hospital stay, and patients undergoing general anesthesia stay longer in hospital.

 In conclusion, there are no studies including large series about foreign body aspirations from tracheostomy. Therefore, there is no consensus on the treatment approach in foreign body aspirations from tracheostomy. Although rigid and FOB each have advantages and disadvantages compared to each other in foreign body removal, according to our experience, the operation time of patients who undergo FOB is shorter than rigid bronchoscopy and they are discharged earlier, although it is not statistically significant. In addition, the fact that FOB does not require general anesthesia prevents complications that can occur due to anesthesia in elderly patients with many additional diseases. In our opinion, FOB should be preferred in patients who aspirate foreign body from tracheostomy. Furthermore, all bronchoscopic procedures should be performed under operating room conditions.
